# On-Chip Non-Dispersive Infrared CO_2_ Sensor Based on an Integrating Cylinder †

**DOI:** 10.3390/s19194260

**Published:** 2019-09-30

**Authors:** Xiaoning Jia, Joris Roels, Roel Baets, Gunther Roelkens

**Affiliations:** 1 Photonics Research Group, INTEC, Ghent University-imec, Technologiepark 126, 9052 Gent, Belgium; 2 Center for Nano- and Biophotonics, Ghent University, 9000 Gent, Belgium; 3 Melexis Technologies NV, Transportstraat 1, 3980 Tessenderlo, Belgium

**Keywords:** optical sensor, CO_2_ sensor, non-dispersive infrared (NDIR), silicon photonics

## Abstract

In this paper, we propose a novel, miniaturized non-dispersive infrared (NDIR) CO_2_ sensor implemented on a silicon chip. The sensor has a simple structure, consisting of a hollow metallic cylindrical cavity along with access waveguides. A detailed analysis of the proposed sensor is presented. Simulation with 3D ray tracing shows that an integrating cylinder with 4 mm diameter gives an equivalent optical path length of 3.5 cm. The sensor is fabricated using Deep Reactive Ion Etching (DRIE) and wafer bonding. The fabricated sensor was evaluated by performing a CO_2_ concentration measurement, showing a limit of detection of ∼100 ppm. The response time of the sensor is only ∼2.8 s, due to its small footprint. The use of DRIE-based waveguide structures enables mass fabrication, as well as the potential co-integration of flip-chip integrated midIR light-emitting diodes (LEDs) and photodetectors, resulting in a compact, low-power, and low-cost NDIR CO_2_ sensor.

## 1. Introduction

CO_2_ gas sensing is receiving increasing attention in both industry and academia, due to its widespread applications such as in air-quality monitoring [1], greenhouse farming [2] and industrial process control [3]. The European advanced CO_2_ sensor market is expected to grow to 3.6 billion dollars by 2021 with a compound annual growth rate of 14.6% [4], which is a huge market for low-cost, miniaturized CO_2_ sensors. Moreover, the European Union introduced a gradual ban on the usage of fluorinated gases in mobile air conditioning systems (MAC) for environmental and fire safety reasons [5]. CO_2_ is considered to be a suitable substitute for MAC refrigerants, as its global warming potential (GWP) is several thousand times smaller than that of fluorinated gases [6]. However, safety concerns about potential leakage of CO_2_, e.g., on board of a car, arise. As the vehicle cabin is a small-volume, relatively confined environment, a trivial leakage of CO_2_ on board may lead to fatal situations for the driver and the passengers. It has been shown that the CO_2_ concentration in a car can increase up to 7% within one minute when a sudden leak happens [7]. Although CO_2_ is not classified as a toxic gas, exposure to elevated CO_2_ concentration levels can produce a variety of health problems. Concentrations of 7% can cause headache, dizziness, visual disturbances, and even unconsciousness within a few minutes [8]. This can be very dangerous for the people on board as well as for the traffic. CO_2_-related symptoms can also occur at much lower concentration levels. Studies have shown that at concentrations as low as 1000 ppm (only about 3 times higher than the typical outdoor concentration), an exposure time of 2.5 h can lead to decline in cognitive abilities and basic activity level [9,10]. The colorless and odorless nature of CO_2_ gas make it practically impossible to detect with the human senses, and therefore sensors are needed to constantly monitor the CO_2_ concentration in order to remove the safety concerns. Different applications may differ in their requirements of sensor performance such as detection limit, accuracy and response time. For air-quality monitoring and greenhouse farming, a detection limit and accuracy of sub-100 ppm is needed, for leak detection in mobile air conditioning systems, a detection limit of a few thousands ppm is sufficient, yet a fast response time is essential since the CO_2_ concentration in the cabin increases quite rapidly as previously discussed. In this work, we are targeting both applications by demonstrating a fast sensor with low limit of detection.

The existing CO_2_ sensors can be categorized into 2 types: electrochemical sensors and non-dispersive infrared (NDIR) sensors. Electrochemical sensors measure the CO_2_ concentration by measuring a change in the electrical properties of materials induced by the CO_2_ adsorption. They have the advantage of being low cost and compact [11,12,13,14]. However, electrochemical sensors suffer from short-term stability, low durability and cross-response to other gases (e.g., water vapor) [15,16]. In contrast, NDIR sensors offer long-term stability, high accuracy and high gas specificity [17], and this method is more favorable compared to electrochemical sensors as CO_2_ is an inert gas and has minimal electrochemical response. Typically, NDIR CO_2_ sensors use the strong absorption of CO_2_ around 4.25 µm, a wavelength range where no other common molecules absorb, thereby avoiding cross-sensitivity issues. Due to these advantages, 83% of the European advanced CO_2_ sensor market uses NDIR sensors [4]. However, NDIR sensors tend to be bulky as a long (typically several cm) interaction length is required to achieve ppm level detection [18,19], and they are also expensive as they are based on discrete co-assembled optical elements, which limit their application in price and size sensitive markets. Intense efforts have been made to miniaturize NDIR sensors, mainly by optimizing the optics design [20,21,22,23], or by using optical cavities [24,25]. Furthermore, pre-concentrators have been employed in NDIR sensors to effectively enhance the CO_2_ concentration and thus decrease the required optical path length [26,27].

In this paper, we present a continuation and extension of our work [28] published in Optical Sensors and Sensing Congress—OSA 2019. This extension demonstrates an NDIR CO_2_ sensor based on an integrating cylinder implemented on a silicon substrate. The sensor consists of a hollow gold-coated cylindrical cavity along with hollow metallic waveguides, one at the input and two at the output, realized by Deep Reactive Ion Etching (DRIE) and wafer bonding. The design of the sensor is inspired by the concept of an integrating sphere. In an integrating sphere, the incident light experiences multiple reflections before it reaches the detector, which effectively increase the optical path length. Here, we have taken the concept of the integrating sphere into the world of silicon photonics, by realizing it using MEMS-compatible fabrication technologies. The use of an integrating cylinder allows for a compact sensor with a long interaction length. Simulation with 3D ray tracing shows the sensor has an equivalent path length of ∼3.5 cm, with a footprint of only 6 × 6 mm^2^. CO_2_ sensing experiments are carried out on the sensor, showing a detection limit of ∼100 ppm. Moreover, the sensor shows a response time of only 2.8 s. The fast response of the sensor is due to its small size for gas to diffuse.

## 2. NDIR Working Principle

Many gas molecules have specific vibrational and rotational absorption lines in the mid-IR range [29], and this unique absorption is often used to detect their presence and concentration. It is well known that CO_2_ has a strong absorption band around a wavelength of 4.25 µm, and the absorption is quantitatively described by the well-known Beer–Lambert law (for monochromatic light):

(1) $$I=I_{0}e^{-\epsilon cL}$$

where *I* and $I_{0}$ are the light intensity at the output and input, respectively, ϵ is the molar attenuation coefficient, *c* is the CO_2_ concentration, and *L* is the interaction length. The transmission from the source to the detector is thus given by:

(2) $$T=\frac{I}{I_{o}}=e^{-\epsilon cL}$$

**Figure 1:**
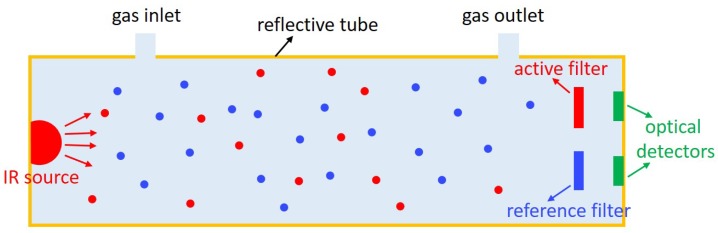
Schematic of a typical NDIR sensor. The sensor consists of an infrared broadband source, a reflecting gas tube with gas inlet and outlet, a pair of optical filters(active and reference), and two optical detectors. The two filters and two detectors form the active channel and reference channel, respectively.

One can see that for a fixed sensor configuration(fixed *L*), the transmission is related to the CO_2_ concentration, thus by measuring the transmission change one can deduce the CO_2_ concentration in the gas sample. The structure of a typical NDIR CO_2_ sensor is shown in Figure 1, which consists of four elements: an optical source at and around 4.25 µm, a gas chamber, optical bandpass filters, and detectors. The active filter is typically centered at 4.25 µm where CO_2_ has strong absorption (active channel), and the reference filter is typically centered at 3.9 µm, where no common gas molecules absorb (reference channel). During a CO_2_ sensing measurement, the signal at the active channel ($I_{A}$) will experience exponential decay due to CO_2_ absorption, while the signal in the reference channel ($I_{R}$) will not change. Thus, by comparing the signal in both channels the CO_2_ concentration in the target gas can be calculated. The presence of the reference channel is to eliminate the impact of source fluctuation, assuming it has the same influence on both the active and reference channels.

## 3. Sensor Design

**Figure 2:**
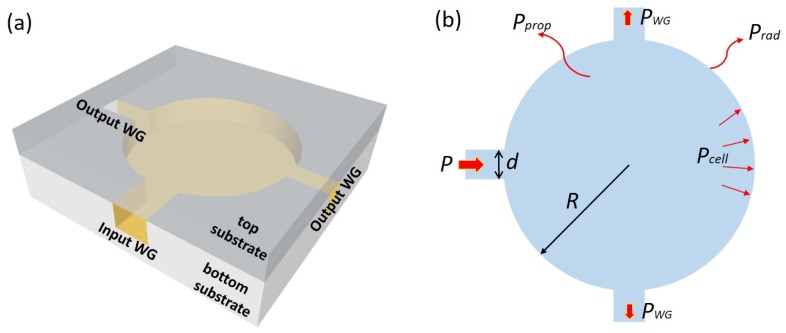
(**a**) 3D schematic of the proposed sensor structure. The bottom substrate has a gold-coated hollow cylindrical cavity with one input waveguide and two output waveguides, the upper substrate is a gold-coated planar silicon substrate, the sensor is formed by wafer bonding of both substrates. (**b**) 2D schematic of the sensor, where *R* is the radius of the circle, *d* is the width of all access waveguides, *P* is the power coupled into the cavity, $P_{WG}$, $P_{rad}$ and $P_{prop}$ are the power coupled to the output waveguides, the power absorbed/radiated at the cavity sidewall, and the power lost during propagation in the cavity, respectively.

Figure 2a shows the 3D schematic of the sensor proposed in this work. It consists of a gold-coated hollow cylindrical cavity with one input waveguide and two output waveguides. The gold-coated inner surface acts as a reflector to confine light inside the cavity, the input waveguide is used to launch incident light into the integrating cylinder and the output waveguides are used to extract the light signal from the cavity and couple it to detectors. With external optical filters, the two output waveguides can act as active channel and reference channel for the sensor. On the cavity sidewall, random roughness is deliberately applied to scatter the incident light into random directions. As the incident light experiences multiple reflections before reaching the detector, a long effective path length can be achieved inside the cylindrical cavity.

In the cavity slab, the incident light is just specularly reflected on the parallel gold-coated top and bottom surfaces. Therefore, the analysis of the integrating cylinder can be reduced from 3D to 2D, as shown in Figure 2b. When we assume that the light incident on the sidewall is reflected in a random direction, the average distance the light beam travels between two consecutive reflections, is given by [30]:

(3) $$L_{avg}=\frac{4}{\pi }\cdot R$$

with *R* being the radius of the circle. When incident light with power *P* is coupled into the cavity through the input waveguide, at steady state, there is a uniform distribution of energy in the cavity. When we define the power incident on the sidewall as $P_{cell}$, then the total input power *P* coupled to the cavity can be “lost” through the extraction loss from each of the access waveguides, the radiation loss/absorption upon incidence on the sidewall and the propagation loss through the cavity slab, given by

(4) $$P_{WG}=\beta P_{cell}$$

(5) $$P_{rad}=\gamma P_{cell}$$

(6) $$P_{prop}=\eta P_{cell}$$

respectively [31]. β is the extraction coefficient from the access waveguides, and is given by the ratio of the width of the waveguide (*d*) and the circumference of the circle:

(7) $$\beta =\frac{d}{2\pi R}$$

γ is the fraction of power radiated/absorbed at the sidewall, related to the reflectivity ρ of the sidewall mirror:

(8) γ=1−ρ

η is the loss of the light propagating in the cavity slab between two consecutive reflections on the sidewalls, whether by the waveguide loss $\alpha_{prop}$ or by the CO_2_ absorption, characterized by the absorption coefficient $\alpha_{CO2}$:

(9) $$\begin{array}{cc} \eta & =1-\exp(-L_{avg}\left(\alpha_{prop}+\alpha_{CO_{2}}\right))\\ \; & \approx L_{avg}\left(\alpha_{prop}+\alpha_{CO2}\right) \end{array}$$

Due to energy conservation, the input power equals the summation of all losses:

(10) $$P=3P_{WG}+P_{prop}+P_{rad}$$

From Equations (4)–(6) and Equation (10) we can deduce a relationship between the fraction of power coupled to the waveguide $P_{WG}$ and the input power *P*:

(11) $$P_{WG}=\frac{\beta }{3\beta +\gamma +\eta }P$$

Thus, the transmission from the input to the output waveguide is given by:

(12) $$\begin{array}{cc} T & =\frac{P_{WG}}{P}=\frac{\beta }{3\beta +\gamma +\eta }\\ \; & =\frac{\beta }{3\beta +1-\rho +\frac{4}{\pi }R\left(\alpha_{prop}+\alpha_{CO2}\right)} \end{array}$$

where the term $\frac{4}{\pi }R$ is the average distance between two consecutive reflections as shown in Equation (3). We define the sensitivity *S* of the sensor to be the transmission change induced by CO_2_ absorption, a quantity that can be calculated by taking the partial derivative of the transmission *T* w.r.t $\alpha_{CO2}$:

(13) $$S=-\frac{\partial T}{\partial \alpha_{CO2}}=\frac{\frac{2}{\pi }\beta R}{{\left(3\beta +1-\rho +\frac{4}{\pi }R\left(\alpha_{prop}+\alpha_{CO2}\right)\right)}^{2}}$$

which has a maximum as a function of *R* (assuming a fixed β) when

(14) $$3\beta +1-\rho =\frac{4}{\pi }R\left(\alpha_{prop}+\alpha_{CO2}\right)$$

The term on the left-hand side of Equation (14) can be considered to be the “extraction” from the cavity sidewall, which consists of extraction by the waveguides and the radiation/absorption loss from the sidewalls as the reflectivity ρ is less than 1. The term on the right-hand side is the absorption in the slab of the cavity, which consists of waveguide loss and CO_2_ absorption loss. It can be seen from Equation (13) that the sensor is most sensitive when the “extraction” equals “absorption”, i.e., when critical coupling occurs inside the cavity. The optimal radius can be calculated by solving Equation (14), which gives:

(15) $$R_{opt}=\frac{3\beta +1-\rho }{\frac{4}{\pi }\left(\alpha_{prop}+\alpha_{CO_{2}}\right)}$$

Since 1−ρ<<3β in our design (β is of the order 0.03, while the reflectivity ρ is of the order 0.995 [32]), by substituting Equation (15) into Equation (12) we find the total transmission loss from the input to the output at optimal radius:

(16) $$\begin{array}{ccc} loss(\mathrm{dB}) & = & -10\log T\\ \; & = & -10\log(\frac{\beta }{2(3\beta +1-\rho )})\\ \; & \approx & 10\log6\\ \; & = & 7.8\mathrm{dB} \end{array}$$

One can see that the total transmission loss is constant at the optimal cylinder radius. This is because at critical coupling, “absorption” equals “extraction”, adding 3 dB to the loss, and in the “extraction” part, power is equally coupled into 3 access waveguides, which gives another 4.8 dB loss (33%), so the total transmission loss is 7.8 dB at the optimal radius assuming a fixed β. In order to calculate the equivalent optical path length, i.e., the effective path length of light from the input waveguide to the output waveguide, we write the transmission from the input to the output with/without CO_2_ as:

(17) $$\begin{array}{ccc} T_{prop+CO_{2}} & = & \frac{1}{3}\exp\left(-\left(\alpha_{prop}+\alpha_{CO_{2}}\right)L_{eq}\right)\\ T_{prop} & = & \frac{1}{3}\exp\left(-\alpha_{prop}L_{eq}\right) \end{array}$$

The factor $\frac{1}{3}$ is because the extracted power is equally coupled into 3 access waveguides. We take the ratio on both sides of Equation (17), and since $\alpha_{CO_{2}}L_{eq}<<1$, in first order approximation:

(18) $$\begin{array}{ccc} \frac{T_{prop+CO_{2}}}{T_{prop}} & = & \exp(-\alpha_{CO_{2}}L_{eq})\\ \; & \approx & 1-\alpha_{CO_{2}}L_{eq} \end{array}$$

On the other hand, $\frac{T_{prop+CO_{2}}}{T_{prop}}$ can be also calculated from Equation (12),

(19) $$\frac{T_{prop+CO_{2}}}{T_{prop}}=\frac{3\beta +1-\rho +\frac{4}{\pi }R\alpha_{prop}}{3\beta +1-\rho +\frac{4}{\pi }R\left(\alpha_{prop}+\alpha_{CO2}\right)}$$

The equivalent path length $L_{eq}$ can thus be calculated by equating Equations (18) and (19):

(20) $$L_{eq}=\frac{\frac{4}{\pi }R}{3\beta +1-\rho +\frac{4}{\pi }R\left(\alpha_{prop}+\alpha_{CO_{2}}\right)}$$

We notice that the numerator is the average path length between two consecutive reflections given by Equation (3), and the denominator is the summation of the coefficients of all losses. We substitute Equation (15) into Equation (20) and we can calculate the equivalent path length at optimal radius:

(21) $$L_{eq,opt}=\frac{1}{2(\alpha_{prop}+\alpha_{CO_{2}})}$$

## 4. Simulations

We used a 3D ray tracing software package Zemax(OpticStudio 16.5, version October 2016, Zemax, Seattle, WA, USA) to simulate the proposed sensor structure. The non-sequential system module was used, allowing for light to be reflected multiple times at a certain interface. In the simulation, we constructed a cylindrical cavity with one input waveguide and two output waveguides, the height of the cavity and the access waveguides were fixed at 300 µm, and the length of the access waveguides was 1 mm. A gold coating was applied on the cavity as well as on the waveguide to model the reflector. An isotropic point source was placed at the entrance of the input waveguide, emitting monochromatic light at 4.25 µm. A total of 100,000 rays was launched into the input waveguide. A rectangular detector with the same dimensions as the cross-section of the access waveguide was placed at one of the output waveguides. To model the scattering at the sidewall, we implemented Lambertian scattering on the cavity sidewall, in which 1 incident ray was scattered into 5 rays, with scattering directions following a Lambertian distribution. To simulate the equivalent path length of the sensor, we used an absorbing medium with an absorption coefficient $\alpha_{CO2}$ to model absorption of CO_2_. From Equation (18), the equivalent path length can be calculated by:

(22) $$L_{eq}\left(cm\right)=-\ln\frac{T_{prop+CO_{2}}}{T_{prop}}/\alpha_{CO2}$$

where $T_{prop+CO_{2}}$ and $T_{prop}$ are the transmission from the input waveguide to the output waveguide with/without the absorbing medium, respectively.

**Figure 3:**
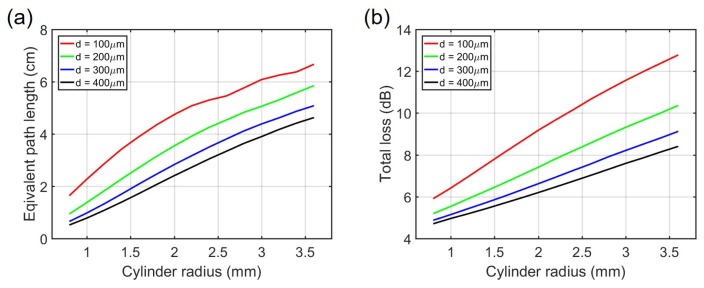
Simulation of the integrating cylinder with various cylinder radius and waveguide width combinations, the height of the cylinder is kept at 300 µm. (**a**) Equivalent path length. (**b**) Total transmission loss.

Figure 3 shows the equivalent path length as well as the total loss from the input waveguide to the output waveguide for various cavity radii *R* and access waveguide widths *d*. In this simulation we used $\alpha_{CO2}$ = 0.03/cm, which is equivalent to the absorption of 1000 ppm CO_2_ averaged over 4.2–4.35 µm.

One can see that as the cavity radius increases or the access waveguide width decreases, the equivalent path length increases. This is because light is trapped inside the cavity for a longer time and has a lower probability to escape to the access waveguides, resulting in a longer equivalent path length. Consequently, light experience more reflections on average and thus the total loss increases.

**Figure 4:**
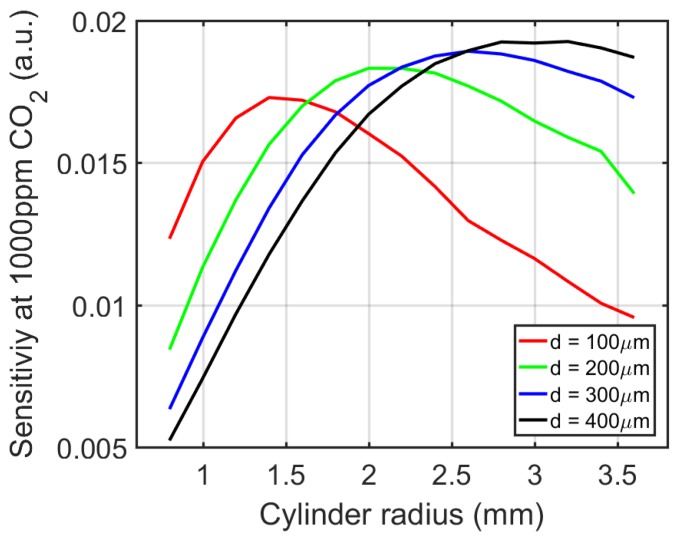
Sensor sensitivity *S* at CO_2_ = 1000 ppm, for various cylinder radius and access waveguide width combinations.

To optimize the design, namely to maximize the sensitivity *S* (i.e., transmission change for a CO_2_ concentration change of 1000 ppm) induced by the CO_2_ absorption, the sensitivity *S* for various cylinder radii and access waveguide widths is plotted in Figure 4.

We can see that as we increase the cylinder radius *R* with fixed access waveguide width *d*, the sensitivity *S* first increases and then decreases. The optimal radius *R* is defined as the radius when *S* is at its maximum. Table 1 summarizes the optimal radius for different access waveguide widths. We can also see that at optimal cylinder radius, both the total loss and the equivalent path length extracted from the simulations are nearly constant, which agrees with theory. As a tradeoff between maximum response and device footprint, we choose a sensor with cylinder radius R=2 mm and access waveguide width d = 200 µm. Such a sensor gives an equivalent path length of 3.5 cm, which is considerable given the small footprint of the sensor.

## 5. Sensor Fabrication and Experimental Setup

### 5.1. Sensor Fabrication

**Figure 5:**
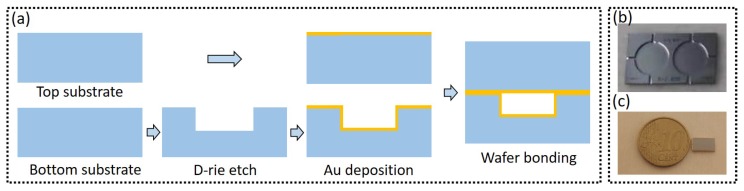
(**a**) Fabrication process of the sensor. Before wafer bonding, the two substrates undergo Ar plasma treatment. (**b**) Two cylindrical cavities etched on the bottom substrate with DRIE, the etching depth is 300 µm, the length of the access waveguides is 1mm. (**c**) Fabricated chip with a EURO 10 cent coin, the chip contains two sensors.

Figure 5a shows the fabrication process of the sensor. To make the sensor on-chip, we started with two blank Si substrates. On the bottom substrate, a cylindrical cavity with one input waveguide and two output waveguides was etched by Deep Reactive Ion Etching (DRIE), as shown in Figure 5b. On the circular boundary of the cavity, a roughness function was deliberately applied, such that the incident light is scattered randomly. After etching, a thin layer of titanium and gold(Ti/Au∼30 nm/500 nm) was sputtered on the bottom substrate as well as on the (planar) top substrate. The reason we used gold as reflector is that it has very high(∼99.5%) reflectivity in the 4 µm wavelength range [32]. After gold deposition, the two substrates underwent an Ar plasma treatment (pressure = 53 mTorr, power = 100 W, duration = 60 s), and were bonded using gold-to-gold direct bonding (at 300 °C, 1 MPa for 30 min). Figure 5c shows a Si chip after bonding, the chip consists of 2 sensors.

### 5.2. Measurement Setup

**Figure 6:**
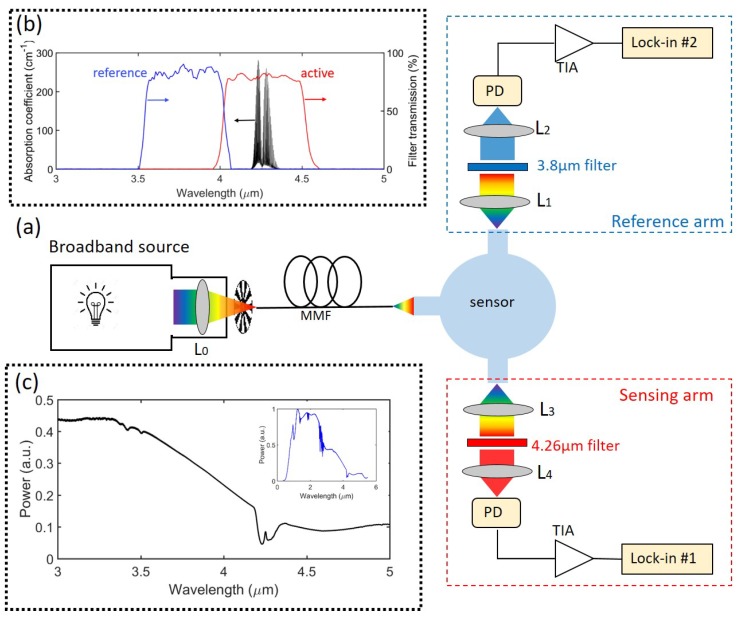
(**a**) Experimental setup. L: lens MMF: multimode fiber, PD: photodiode, TIA: trans-impedance amplifier. (**b**) Transmission spectrum of the optical filters used in this work(reproduced from [33]), superimposed on the CO_2_ absorption spectrum in 3–5 µm wavelength range [29]. (**c**) Source spectrum in 3–5 µm wavelength range, a full spectrum is also in the inset, also reproduced from [33].

To experimentally evaluate the fabricated sensor, we built the setup as shown in Figure 6. The light emitted from a stabilized broadband source (SLS202, with SLS202C collimation package, Thorlabs, Newton, NJ, USA) is passed through a lens(LA5315-E, Thorlabs, Newton, NJ, USA) and focused onto a multimode fiber (CIR500/550, Artphotonics, Berlin, Germany). An optical chopper (MC1F10 blade with MC2000 controller, Thorlabs, Newton, NJ, USA) is placed between the lens and fiber to modulate the continuous wave light. Then light is butt-coupled from the fiber to the input waveguide of the sensor (fiber to input waveguide distance ∼50 µm). At the output waveguide of the sensing arm, light is collimated and re-focused onto a photodiode by two identical lenses (C037TME-E, Thorlabs, Newton, NJ, USA), with a band pass filter (FB4250-500, Thorlabs, Newton, NJ, USA) in between. The lens-filter-lens system is sealed and isolated from the ambient to avoid excess absorption loss by a CO_2_ concentration change in the gap between lenses and filter. We use a commercial un-cooled photodiode (P13023-013CA, Hamamatsu, Japan) as a detector. A customized trans-impedance amplifier (TIA) is designed and fabricated in-house to amplify the signal. A lock-in amplifier (SR830, Stanford Research Systems, Sunnyvale, CA, USA) is used for data acquisition and readout. The reference arm only differs in the optical filter, with pass band centered at $\lambda_{0}$ = 3.75 µm (FB3750-500, Thorlabs, Newton, NJ, USA), the two arms are otherwise identical. The transmission spectra of the active and reference filters are shown in Figure 6b. CO_2_ gas was supplied from certified cylinders (PRAXAIR Gases, Danbury, CT, USA). Sample gas with various concentrations of CO_2_ is generated by mixing CO_2_ and N_2_ through two mass flow controllers (M13212646C, Bronkhorst, Netherlands), and the sample gas is fed into the sensor by bringing the gas tube in close proximity to the sensor (not shown in the setup). During the measurement, the optical chopper works at 635 Hz, and the signals at both arms are simultaneously acquired by two lock-in amplifiers with identical settings.

## 6. Experimental Results

As mentioned before, the reference channel is used to compensate for source fluctuations. The source power fluctuations can be eliminated by constantly measuring the reference channel intensity $I_{R}$ with a correction factor $\frac{I_{R0}}{I_{A0}}$, which can be obtained by flushing the sensor with CO_2_-free air at the beginning of the measurement. The normalized signal can thus be expressed as:

(23) $$S_{norm}=1-\frac{I_{R0}}{I_{A0}}\frac{I_{A}}{I_{R}}$$

In all following measurement results, we use the quantity $S_{norm}$ as a figure of merit to evaluate the sensor performance.

### 6.1. Allan Deviation Plot

**Figure 7:**
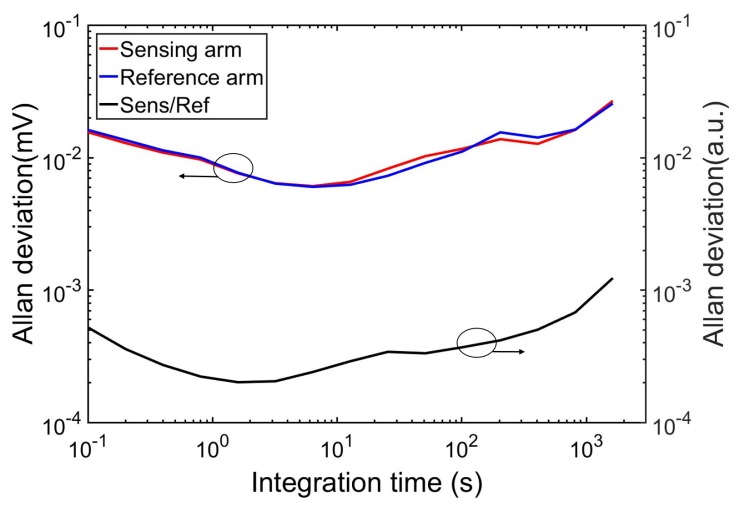
Allan deviation plot of the sensing and reference arm, as well as the normalized signal (in pure N_2_ environment).

As the limit of detection (LOD) of the sensor is limited by the system noise, an Allan deviation analysis was carried out to study both system stability and theoretical LOD of the sensor. During the measurement, we flush the sensor with pure nitrogen and record the sensing signal and reference signal. The Allan deviation of both sensing signal $I_{A}$ and reference signal $I_{R}$, as well as the normalized signal $\frac{I_{A}}{I_{R}}$, are presented in Figure 7. One can see that the optimal averaging time (when the Allan deviation of the normalized signal is at its minimum), is approximately 2 s, and the minimum transmission change that can be measured is 0.0002 (1 σ). In all following measurements an integration time of 1 second was used, which is restricted by the limited resolution of the lock-in time constant in the experiment.

### 6.2. CO_2_ Response

**Figure 8:**
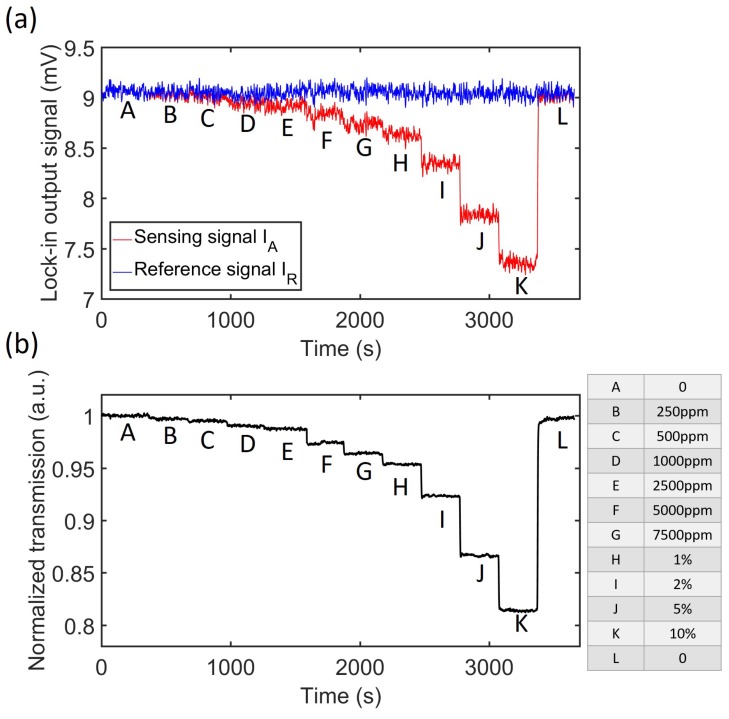
(**a**) Response of both sensing signal and reference signal to CO_2_ concentration steps, the sensing and reference signal exhibit a strong correlation. (**b**) Normalized transmission, obtained by dividing the sensing signal with the reference signal. The CO_2_ concentration steps are listed on the right side.

**Figure 9:**
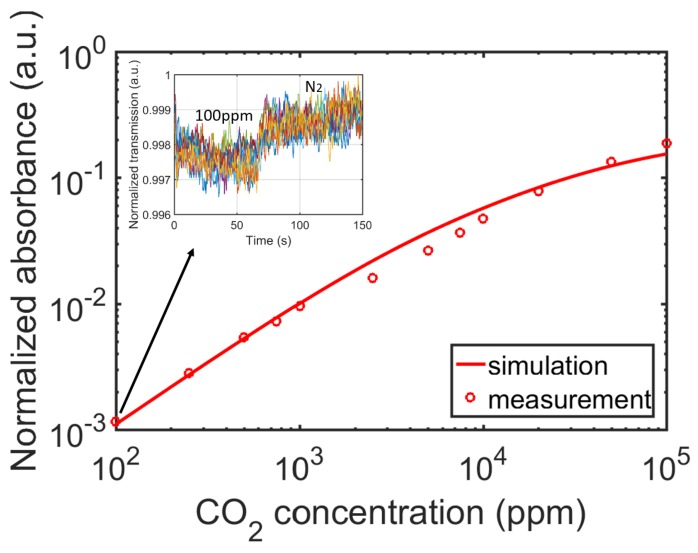
Normalized absorbance of the sensor at various CO_2_ concentrations. The inset shows the step response of the sensor at CO_2_ = 100 ppm, with 10 measurements superimposed in one figure.

To measure the response of the sensor to CO_2_ concentration change, a series of sample gases with different CO_2_ concentrations were generated and fed into the sensor. At each concentration step, the sensing signal $I_{A}$ and the reference signal $I_{R}$ were recorded for a period of 5 min, as shown in Figure 8a. It can be seen that the reference signal stays relatively stable while the sensing signal responds to CO_2_ concentration changes. Moreover, a strong correlation can be observed between the two signals, due to the common mode noise on both arms. These fluctuations can be eliminated by calculating the normalized signal $\frac{I_{A}}{I_{R}}$, shown in Figure 8b, with the CO_2_ concentration sequence listed on the right side. According to Equation (6), we can calculate the normalized signal $S_{norm}$ for each CO_2_ concentration. The values of $I_{R0}$ and $I_{A0}$ are obtained by flushing the sensor with pure nitrogen at the beginning of the measurement. The calculated normalized absorbance is plotted in Figure 9. We also simulated the absorbance by propagating the source spectrum (shown in Figure 6c) through the active filter (shown in Figure 6b) and then through CO_2_, using an equivalent path length of 3.5 cm (as obtained from Figure 3a). One can see that the simulation and the measurement agree reasonably well. The inset in Figure 9 shows the normalized signal $\frac{I_{A}}{I_{R}}$ of 100 ppm CO_2_, where we flushed the sensor first with 100 ppm CO_2_ and then with pure nitrogen, the inset shows 10 such measurements superimposed over one figure. A clear and repeatable step response is observed at 100 ppm CO_2_.

### 6.3. Response Time

**Figure 10:**
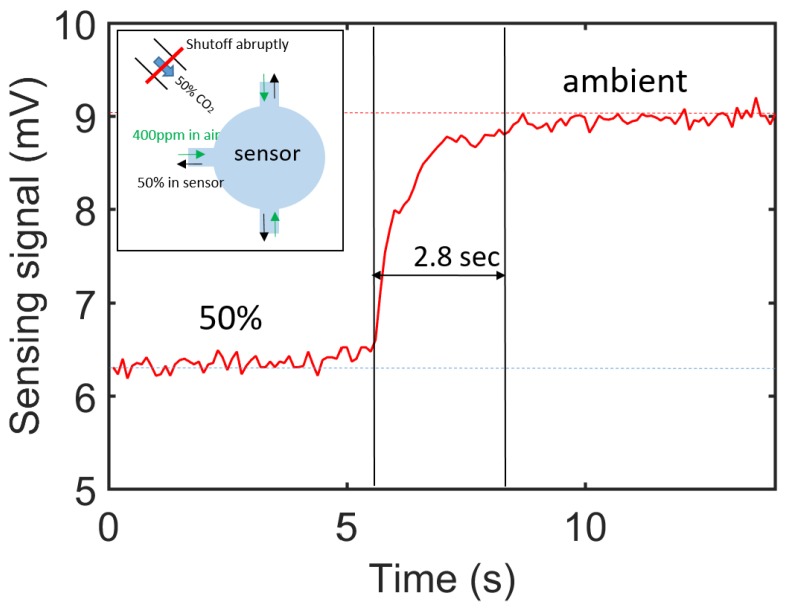
Response time of sensor, measured with 100 ms lock-in integration time. The inset shows the gas flow mechanisms when the 50% CO_2_ is abruptly shut off.

Response time is an important characteristic of the sensor, as fast response time enables real time detection, which is of great importance for applications such as in MAC leak detection. Response time is defined as the time needed for the sensor to reach 90% of the total response when there is a step concentration change. To measure the response time of the sensor, we used the same experimental setup as shown in Figure 6a, the measurement method is shown in the inset of Figure 10. We first flushed the sensor with a sample gas containing 50% of CO_2_, and recorded the sensing signal when the reading became stable. Then we abruptly shut off the gas flow such that the CO_2_ concentration reaches ambient level (∼400 ppm). The measured time trace sensing signal is shown in Figure 10. It can be seen that the response time (from 10% to 90%) of the sensor is approximately 2.8 s, due to the sensor’s small footprint.

## 7. Conclusions and Outlook

In summary, a miniaturized CO_2_ sensor based on the NDIR working principle is demonstrated in this work. The sensor has a gold-coated cylindrical cavity with one input waveguide and two output waveguides, in which light experiences multiple reflections and as such the optical path length is effectively increased. The design of the sensor is optimized by simulations with 3D ray tracing. We show that with an access waveguide width of 200 µm and a cylinder radius of 2 mm, the sensor can have an equivalent path length of 3.5 cm on a footprint of only 6 mm × 6 mm. The sensor was fabricated using DRIE and wafer bonding on silicon. To characterize the sensor, CO_2_ sensing measurements were performed, showing a limit of detection of 100 ppm. The sensor response time was also measured to be 2.8 s. The use of DRIE-based waveguide structures enables mass fabrication, as well as the co-integration of flip-chip integrated mid-IR LEDs and photodetectors to achieve a fully integrated sensing system. Specific performance of the sensor such as long-term stability and cross-sensitivity will be investigated when a fully integrated CO_2_ sensor is available in our future work.

## Figures and Tables

**Table 1:** Optimal cylinder radius for different access waveguide widths, as well as the corresponding equivalent path length and total transmission loss. It can be seen that both the equivalent path length and total transmission loss are nearly constant at optimal radius.

d [um]	R [mm]	TL [dB]	*L_eq_* [cm]
100	1.4	7.5	3.4
200	2.0	7.4	3.5
300	2.6	7.6	3.8
400	3.0	7.6	3.9
